# Hydrocarbon molar water solubility predicts NMDA vs. GABA_A_ receptor modulation

**DOI:** 10.1186/2050-6511-15-62

**Published:** 2014-11-19

**Authors:** Robert J Brosnan, Trung L Pham

**Affiliations:** Department of Surgical and Radiological Sciences, School of Veterinary Medicine, University of California, Davis, CA 95616 USA

## Abstract

**Background:**

Many anesthetics modulate 3-transmembrane (such as NMDA) and 4-transmembrane (such as GABA_A_) receptors. Clinical and experimental anesthetics exhibiting receptor family specificity often have low water solubility. We hypothesized that the molar water solubility of a hydrocarbon could be used to predict receptor modulation in vitro.

**Methods:**

GABA_A_ (α_1_β_2_γ_2s_) or NMDA (NR1/NR2A) receptors were expressed in oocytes and studied using standard two-electrode voltage clamp techniques. Hydrocarbons from 14 different organic functional groups were studied at saturated concentrations, and compounds within each group differed only by the carbon number at the ω-position or within a saturated ring. An effect on GABA_A_ or NMDA receptors was defined as a 10% or greater reversible current change from baseline that was statistically different from zero.

**Results:**

Hydrocarbon moieties potentiated GABA_A_ and inhibited NMDA receptor currents with at least some members from each functional group modulating both receptor types. A water solubility cut-off for NMDA receptors occurred at 1.1 mM with a 95% CI = 0.45 to 2.8 mM. NMDA receptor cut-off effects were not well correlated with hydrocarbon chain length or molecular volume. No cut-off was observed for GABA_A_ receptors within the solubility range of hydrocarbons studied.

**Conclusions:**

Hydrocarbon modulation of NMDA receptor function exhibits a molar water solubility cut-off. Differences between unrelated receptor cut-off values suggest that the number, affinity, or efficacy of protein-hydrocarbon interactions at these sites likely differ.

## Background

Inhaled anesthetics interact with putative cell receptor targets in a manner uncharacteristic of most other pharmacologic agents. They exhibit immobilization efficacy in all animals, both vertebrates and invertebrates, and even in protozoa and plants. The inhaled agents range in diversity from single elements to diatomic molecules to complex hydrocarbons and share no conserved size, shape, or functional groups. Most agents also modulate multiple phylogenetically unrelated cell targets and generally inhibit excitatory channels or receptors and potentiate inhibitory ones, complicating the identification of a drug-receptor structural motif predictive of anesthetic molecular action [1].

Attempts to determine how disparate drugs act on so many unrelated receptors—and to define those targets essential to inhaled anesthetic actions—have thus far proved illusory. Consequently, in the present study, we posed the opposite question: Why are some anesthetics unable to modulate certain anesthetic-sensitive ion channels or receptors, even at supra-pharmacologic concentrations? To answer this question, we restricted consideration to two unrelated ion channels in particular: the N-methyl-d-aspartate (NMDA) receptor, a member of the 3-transmembrane (TM3) ion channel family, and the γ-aminobutyric acid type A (GABA_A_) receptor, a member of the 4-transmembrane (TM4) ion channel family. Strong evidence supports the role of both receptors in mediating various endpoints of general anesthesia, including immobility and amnesia [2–6]. Within the NMDA and GABA_A_ channel proteins, hydrophilic or amphipathic cavities or pockets have been postulated near the solvent interface and within transmembrane segments of several subunits [7, 8]. Presumably, such cavities might conceivably contain solvent molecules or nothing at all, though it should be thermodynamically less favorable to maintain a vacuum within a pocket that is sufficiently large and polar enough to accommodate water.

The binding affinity of water within each pocket on an ion channel can be expressed as a standard dissociation constant, and the ability of a drug to displace water and either completely or partially fill the pocket can be expressed as a standard equilibrium constant. Since structures of phylogenetically unrelated channels are different, the hydrophilicity of their respective pockets should not be conserved. Therefore, dissociation constants are expected to be different for water bound within pockets of different channels. Consequently, the minimum concentration of drug necessary to displace water within a pocket capable of inducing a functional conformational change should be different between unrelated channels. If the drug cannot reach a concentration within the aqueous phase sufficient to displace water within the protein modulatory pocket, then that drug should exert no pharmacologic effect on the channel, even when delivered at a saturated aqueous phase concentration. The physical property that defines this maximum (saturated) concentration of a drug in the aqueous phase is the molar water solubility.

**Figure 1:**
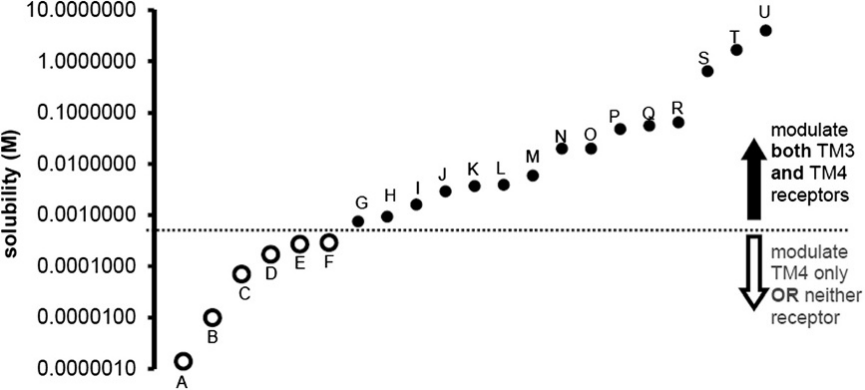
**Summary of ion channel modulation as a function of calculated anesthetic molar solubility in unbuffered water at 25°C (values from SciFinder Scholar).** Drugs that modulate 4-transmembrane receptors (TM4) or neither receptor type are shown as open circles (○, A-F) below the dotted horizontal solubility line. Drugs that modulate both 3-transmembrane (TM3) and TM4 receptors are shown as small black circles (●, G-U) above the dotted horizontal solubility line. A = nonane (unpublished data), B = midazolam [9], C = diazepam [10], D = undecanol [11], E = etomidate [12], F = 1,2-dichlorohexafluorocyclobutane [13], G = sevoflurane [14–17], H = propofol [18, 19], I = ketamine [12, 16, 20], J = isoflurane [14–16, 21, 22], K = enflurane [15, 23], L = dizocilpine [20, 24], M = desflurane [16, 17], N = halothane [14, 22, 23], O = cyclopropane [22, 25], P = chloroform [22], Q = 2,6-dimethylphenol [26], R = methoxyflurane [14, 15, 23], S = diethyl ether [15, 23], T = nitrous oxide [21, 22], U = ethanol [21].

The effects of a number of conventional (modern and historic) and experimental anesthetics on NMDA and GABA_A_ receptors have been studied previously. When plotted in order of their calculated molar water solubilities (Figure 1), there is an abrupt change in the ability of compounds to modulate NMDA receptors. Drugs with water solubility greater than about 1 mM all modulate NMDA and GABA_A_ currents when administered in sufficiently large concentrations. However, drugs with lower molar water solubility modulate only GABA_A_ or neither receptor type. These data suggest that molar water solubility may predict an NMDA receptor cut-off effect at values less than 1 mM and are consistent with non-specific interactions in which water displaced from modulatory pockets on the NMDA receptor contribute, at least in part, to molecular mechanisms of action. Compounds unable to reach approximately 1 mM aqueous concentration may thus be unable to competitively displace water within any of the NMDA critical modulatory sites.

We hypothesized that hydrocarbon molar water solubility below this critical cut-off value is associated with loss of NMDA receptor modulation, but not GABA_A_ receptor modulation. As tests agents, we considered 14 different series of normal hydrocarbon chains or rings, where molecules within each series differed only by the length of the Ω chain or substitutent alkyl groups (Table 1). Because different functional groups differ in hydrophilicity, the length and volume of the hydrocarbon chain needed to achieve a given molar water solubility will likewise differ. Thus, if the hypothesis is correct, then observed cut-off responses should correlate with hydrocarbon molar water solubility rather than hydrocarbon size.

**Table 1:** **Source, purity and physical properties of study compounds**

Compound	CAS#	MW (amu)	P _vap_ (mmHg)	Solubility (M)	Carbon (#)	Volume (Å ^3^)	Source	Purity (%)
Alcohols								
1-decanol	112-30-1	158.28	1.48 × 10^−2^	6.5 × 10^−4^	10	317	Aldrich	>99
1-undecanol	112-42-5		5.10 × 10^−3^	1.7 × 10^−4^	11	344	Acros	98
1-dodecanol	112-53-8	172.31 186.33	2.09 × 10^−3^	4.1 × 10^−5^	12	372	TCI	99
Alkanes								
butane	106-97-8	58.12	1.92 × 10^3^	1.4 × 10^−3^	4	156	Matheson	99.99
pentane	109-66-0	72.15	5.27 × 10^2^	4.3 × 10^−4^	5	184		>99
hexane	110-54-3	86.18	1.51 × 10^2^	1.2 × 10^−4^	6	211	Aldrich Acros	>99
Aldehydes								
octanal	124-13-0	128.21	2.07 × 10^0^	5.4 × 10^−3^	8	262	Aldrich	99
nonanal	124-19-6	142.24	5.32 × 10^−^	2.3 × 10^−3^	9	289	Aldrich	95
decanal	112-31-2	156.27	^1^	9.8 × 10^−4^	10	316	Aldrich	98
undecanal	112-44-7	170.29	2.07 × 10^−1^ 8.32 × 10^−2^	4.2 × 10^−4^	11	344	Aldrich	97
Alkenes								
1-pentene	109-67-1	70.13	6.37 × 10^2^	1.4 × 10^−3^	5	176	Aldrich	99
1-hexene	592-41-6	84.16	1.88 × 10^2^	4.2 × 10^−4^	6	203	Aldrich	>99
Alkynes								
1-hexyne	693-02-7	82.14	1.35 × 10^2^	2.9 × 10^−3^	6	184	Aldrich	97
1-heptyne	628-71-7	96.17	4.35 × 10^1^	6.6 × 10^−4^	7	212	Acros	99
1-octyne	629-05-0	110.2	1.44 × 10^1^	1.9 × 10^−4^	8	239	Acros	99
Amines								
1-octadecanamine	124-30-1	269.51	4.88 × 10^−5^	1.3 × 10^−3^	18	546	TCI	97
1-eicosanamine	10525-37-8	297.56	8.96 × 10^−6^	2.7 × 10^−4^	20	601	Rambus	95
Benzenes								
1,3-dimethylbenzene	108-38-3	106.17	7.61 × 10^0^	1.2 × 10^−3^	8	202	Aldrich	>99
1,3-diethylbenzene	141-93-5	134.22	1.15 × 10^0^	6.6 × 10^−5^	10	257	Fluka	>99
Cycloalkanes								
cyclopentane	287-92-3	70.13	3.14 × 10^2^	3.3 × 10^−3^	5	147	Aldrich	>99
cyclohexane	110-82-7	84.16	9.37 × 10^1^	1.0 × 10^−3^	6	176	Aldrich	>99.7
Ethers								
dibutylether	142-96-1	130.23	7.10 × 10^0^	1.6 × 10^−2^	8	277	Aldrich	99.3
dipentylether	693-65-2		1.00 × 10^0^	3.0 × 10^−3^	10	331	Fluka	>98.5
dihexylether	112-58-3	158.28 186.33	1.48 × 10^−1^	5.8 × 10^−4^	12	386	Aldrich	97
Esters								
ethyl heptanoate	106-30-9	158.24	6.02 × 10^−1^	5.4 × 10^−3^	9	299	MP Bio	99
ethyl octanoate	106-32-1		2.24 × 10^−1^	2.1 × 10^−3^	10	327	Aldrich	>99
ethyl decanoate	110-38-3	172.26 200.32	3.39 × 10^−2^	4.4 × 10^−4^	12	381	TCI	98
Haloalkanes								
1-fluoropentane	592-50-7	90.14	1.84 × 10^2^	3.9 × 10^−3^	5	193	Aldrich	98
1-fluorohexane	373-14-8	104.17	6.06 × 10^1^	1.2 × 10^−3^	6	220	Acros	>99
1-fluoroctane	463-11-6	132.22	7.09 × 10^0^	1.3 × 10^−4^	8	275	Aldrich	98
Ketones								
2-decanone	693-54-9	156.27	2.48 × 10^−1^	3.2 × 10^−3^	10	316	TCI	>99
2-undecanone	112-12-9		9.78 × 10^−2^	1.4 × 10^−3^	11	343	Acros	98
2-dodecanone	6175-49-1	170.29 184.32	3.96 × 10^−2^	5.8 × 10^−4^	12	371	TCI	95
Sulfides								
1-(ethylthio)-hexane	7309-44-6	146.29	8.16 × 10^−1^	2.8 × 10^−3^	8	289	Pfaltz	97
1-(ethylthio)-octane	3698-94-0	174.35	1.08 × 10^−1^	5.0 × 10^−4^	10	344	Pfaltz	97
Thiols								
1-pentanethiol	110-66-7	104.21	1.42 × 10^1^	1.5 × 10^−3^	5	207	Aldrich	98
1-hexanethiol	111-31-9	118.24	4.50 × 10^0^	5.1 × 10^−4^	6	235	TCI	96

Chemical Abstracts Service number (CAS#), molecular weight (MW), vapor pressure at 25°C (P_vap_), molar solubility in pure water at pH = 7, and molecular volume are calculated estimates (rather than measured values) referenced by SciFinder Scholar.

Although generally advocated to study anesthetic effects at clinically-relevant concentrations, we proposed to test this hypothesis at saturated aqueous drug concentrations for 3 reasons. First, because an aqueous binding site is postulated, it is important to study drug effects at an interfacial concentration that can be directly related to a bulk aqueous concentration. However, anesthetics do not distribute equally through the lipid bilayer. Halothane shows a preference for the phospholipid headgroup interface [27]. Xenon atoms prefer regions at the lipid-water interface and the central region of the bilayer [28]. The anesthetics cyclopropane, nitrous oxide, desflurane, isoflurane, and 1,1,2-trifluoroethane all preferentially concentrate at the interface between water and hexane,[29] but the nonimmobilizer perfluoroethane does not exhibit a hydrophilic-hydrophobic interfacial maxima [29]. Nonetheless, even without knowing the membrane distribution profile of an anesthetic, the interfacial concentration can be assumed maximal at a saturating aqueous phase concentration at equilibrium; thus drug responses are compared at their respective relative maximum interfacial concentrations.

Second, different anesthetic endpoints are achieved at different drug concentrations. Thus, a drug could exhibit relative receptor specificity; that is to say, a drug may act preferentially at one receptor to achieve one endpoint—such as amnesia—but act on additional receptors when administered at higher concentrations to achieve other endpoints—such as immobility. Failure to modulate a receptor when a drug is delivered at a saturated concentration implies a null receptor response at lower drug concentrations and for any therapeutic endpoint.

Third, the drug concentrations that produce different anesthetic endpoints—amnesia, unconsciousness and immobility—are unknown for many experimental compounds. Some anesthetic endpoints may not even be achievable with some compounds, such as the nonimmobilizers. Absolute receptor specificity means that there is also relative specificity for any drug effect produced. Accordingly, comparisons of receptor effects using saturated concentrations *in vitro* are not prepositioned upon knowledge of *in vivo* drug anesthetic effects.

Hence, this study aimed to test whether NMDA versus GABA_A_ receptor modulation was correlated with calculated molar water solubility “cut-off” values for diverse series of hydrocarbon functional groups.

## Methods

### Oocyte collection and receptor expression

An ovary from tricaine-anesthetized *Xenopus laevis* frogs was surgically removed using a protocol approved by the Institutional Animal Care and Use Committee at the University of California, Davis (Protocol #12030). Following manual disruption of the ovarian lobule septae, the ovary was incubated in 0.2% Type I collagenase (Worthington Biochemical, Lakewood, NJ) to defolliculate oocytes which were washed and stored in fresh and filtered modified Barth’s solution composed of 88 mM NaCl, 1 mM KCl, 2.4 mM NaHCO_3_, 20 mM HEPES, 0.82 mM MgSO_4_, 0.33 mM Ca(NO_3_)_2_, 0.41 mM CaCl_2_, 5 mM sodium pyruvate, gentamycin, penicillin, streptomycin, and corrected to pH = 7.4. All salts and antibiotics were A.C.S. grade (Fisher Scientific, Pittsburgh, PA).

Clones used were provided as a gift from Dr. R.A. Harris (University of Texas, Austin) and were sequenced and compared to references in the National Center for Biotechnology Information database to confirm the identity of each gene. GABA_A_ receptors were expressed using clones for the human GABA_A_ α1 and the rat GABA_A_ β2 and γ2s subunits in pCIS-II vectors. Approximately 0.25-1 ng total plasmid mixture containing either α_1,_ β_2_, or γ_2_ genes in a respective ratio of 1:1:10 was injected intranuclearly through the oocyte animal pole and studied 2–4 days later. These plasmid ratios ensured incorporation of the γ subunit into expressed receptors, as confirmed via receptor potentiation to 10 μM chlordiazepoxide or insensitivity to 10 μM zinc chloride during co-application with GABA. In separate oocytes, glutamate receptors were expressed using rat NMDA NR1 clones in a pCDNA3 vector and rat NMDA NR2A clones in a Bluescript vector. RNA encoding each subunit, prepared using a commercial transcription kit (T7 mMessage mMachine, Ambion, Austin, TX), was mixed in a 1:1 ratio, and 1–10 ng of total RNA was injected into oocytes and studied 1–2 days later. Oocytes injected with similar volumes of water served as controls.

### GABA_A_ receptor electrophysiology studies

Oocytes were studied in a 250 μL linear-flow perfusion chamber with solutions administered by syringe pump at 1.5 ml/min with gastight glass syringes and Teflon tubing. Oocyte GABA_A_ currents were studied using standard two-electrode voltage clamping techniques at a holding potential of -80 mV using a 250 μL channel linear-flow perfusion chamber with solutions administered by syringe pump at 1.5 mL/min.

Frog Ringer’s (FR) solution composed of 115 mM NaCl, 2.5 mM KCl, 1.8 mM CaCl_2_, and 10 mM HEPES prepared in 18.2 MΩ H_2_O and filtered and adjusted to pH = 7.4 was used to perfuse oocytes. Agonist solutions also contained 20-to-40 μM, equal to an EC_10–20_, of 4-aminobutanoic acid (FR-GABA) [30–32]. After FR perfusion for 5 min, oocytes were exposed to 30 sec FR-GABA followed by another 5 min FR washout; this was repeated until stable GABA_A_-elicited peaks were obtained. Next, FR containing a saturated solution of the study drug (Table 1)—or for gaseous study compounds a vapor pressure equal to 90% of barometric pressure with balance oxygen—was used to perfuse the oocyte chamber for 2 min followed by perfusion with a FR-GABA solution containing the identical drug concentration for 30 sec. FR was next perfused for 5 min to allow drug washout, and oocytes were finally perfused with FR-GABA for 30 sec to confirm return of currents to within 10% of the initial baseline response.

### NMDA receptor electrophysiology studies

Methods for measurement of whole-cell NMDA receptor currents have been described [33, 34]. Briefly, baseline perfusion solutions were the same as for GABA_A_ with the substitution of equimolar BaCl_2_ for calcium salts and the addition of 0.1 mM EGTA; this constituted barium frog Ringer’s solution (BaFR). Agonist solutions for NMDA studies also contained 0.1 mM glutamate (E) and 0.01 mM glycine (G) to constitute a BaFREG solution that produced a NMDA receptor current ≥ EC_99_.

The syringe pump and perfusion chamber apparatus as well as the clamp holding potential and baseline-agonist exposure protocols were identical to that described for the GABA_A_ studies. The same test compounds, concentrations, and preparative methods were used in NMDA voltage clamp studies as in the GABA_A_ voltage clamp studies (Table 1).

### Response calculations and data analysis

Modulating drug responses were calculated as the percent of the control (baseline) peak as follows: $100\cdot \frac{I_{D}}{I_{B}}\text{,}$ where I_D_ and I_B_ were the peak currents measured during agonist + drug and agonist baseline perfusions, respectively. When present, direct receptor activation by a drug was similarly calculated as a percent of the agonist response. Average current responses for each drug and channel were described by mean ± SD. A lack of receptor response (cut-off) was defined as a <10% change from baseline current that was statistically indistinguishable from zero using a two-tailed Student t-test. Hence, drug responses ≥110% of the baseline peak showed potentiation of receptor function, and drug responses ≤90% of the baseline peak showed inhibition of receptor function.

**Figure 2:**
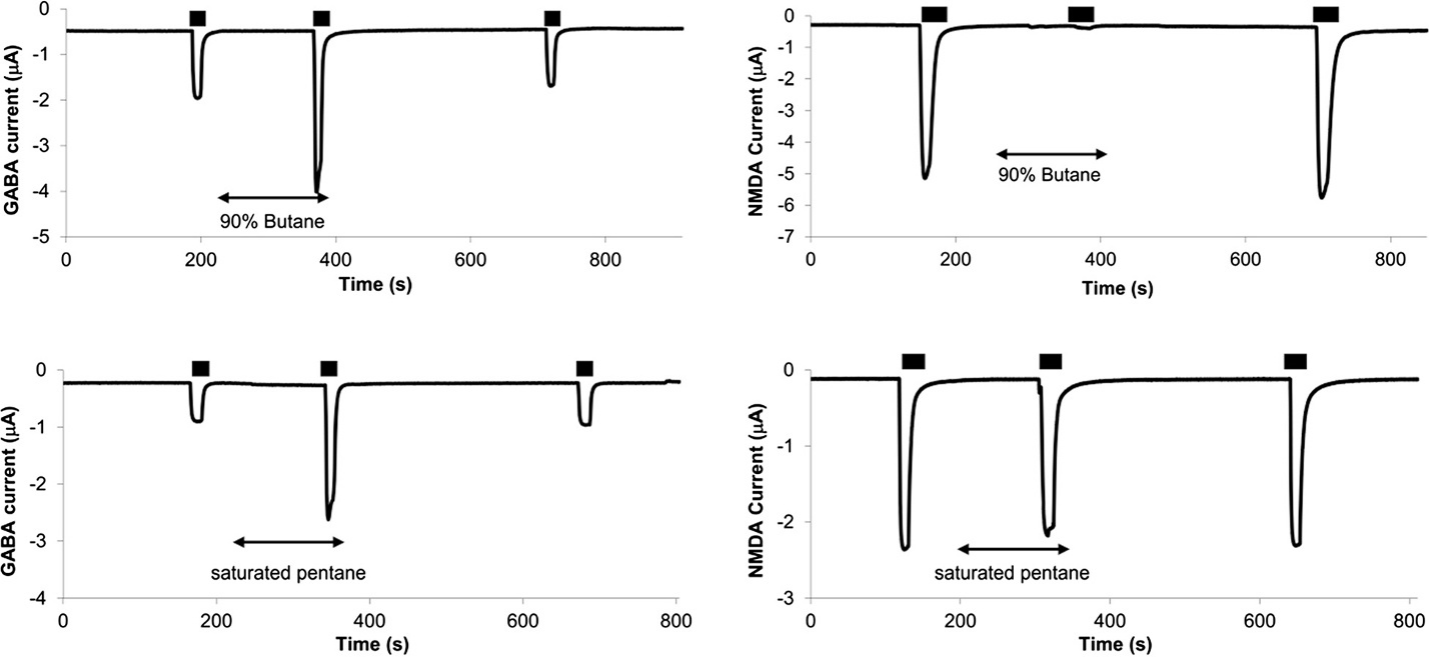
**Sample two-electrode voltage clamp recordings from oocytes expressing either GABA**_**A**_ **receptors (left) or NMDA receptors (right).** Black bars (▬) represent periods of agonist exposure, and arrows (↔) represent periods of saturated alkane exposure. Both butane and pentane positively modulate GABA_A_ receptors. Butane negatively modulates NMDA receptors, but pentane produces no effect. Hence, NMDA receptors exhibit an alkane cut-off between butane and pentane.

The log_10_ of the calculated solubility (log_10_S) for compounds immediately below and above the cut-off for each hydrocarbon functional group were used to determine the receptor cut-off. For each hydrocarbon, there was a “grey area” of indeterminate solubility effect (Figure 2) between sequentially increasing hydrocarbon chain lengths. Mean solubility cut-offs were calculated as the average log_10_S for the least soluble compound that modulated receptor function and the most soluble neighboring compound for which no effect was observed. From this result, a 95% confidence interval for log_10_S was calculated for receptor solubility cut-offs.

## Results

Hydrocarbon effects on NMDA and GABA_A_ receptors are summarized in Table 2, and sample recordings are presented in Figure 2. All of the compounds tested positively modulated GABA_A_ receptor function, and a few of the 5-to-6 carbon compounds caused mild direct GABA_A_ receptor activation, particularly the 1-fluoroalkanes and thiols. Mild direct receptor activation also occurred with dibutylether. With the exception of the aldehydes, alkynes, and cycloalkanes, GABA_A_ receptor inhibition tended to decrease with increasing hydrocarbon chain length. No water solubility cut-off effect was observed for GABA_A_ receptors for the compounds tested.

**Table 2:** **Mean responses (±SEM) produced by 14 different functional groups on NMDA and GABA**_**A**_ **receptor modulation, expressed as a percent of the control agonist peak, using standard two-electrode voltage clamp techniques with 5–6 oocytes each**

Compound	NMDA			GABA _A_		
	% Direct effect	% Agonist effect	Drug response	% Direct effect	% Agonist effect	Drug response
Alcohols						
1-decanol	None	70 ± 3	-	None	386 ± 20	+
1-undecanol	None	101 ± 2	0	None	181 ± 13	+
1-dodecanol	None	98 ± 1	0	None	177 ± 4	+
Alkanes						
butane	None	7 ± 2	-	None	623 ± 68	+
pentane	None	94 ± 3	0	None	321 ± 10	+
hexane	None	100 ± 1	0	None	129 ± 5	+
Aldehydes						
octanal	None	71 ± 3	-	6 ± 3	357 ± 20	+
nonanal	None	104 ± 2	0	None	219 ± 29	+
decanal	None	97 ± 3	0	None	159 ± 5	+
undecanal	None	97 ± 8	0	None	299 ± 29	+
Alkenes						
1-pentene	None	69 ± 1	-	2 ± 3	453 ± 38	+
1-hexene	None	97 ± 0	0	None	132 ± 2	+
Alkynes						
1-hexyne	None	41 ± 6	-	5 ± 2	418 ± 21	+
1-heptyne	None	68 ± 10	-	None	172 ± 8	+
1-octyne	None	96 ± 2	0	None	259 ± 11	+
Amines						
1-octadecanamine	None	73 ± 4	-	None	146 ± 5	+
1-eicosanamine	None	108 ± 1	0	None	166 ± 7	+
Benzenes						
1,3-dimethylbenzene	None	58 ± 3	-	None	366 ± 21	+
1,3-diethylbenzene	None	101 ± 2	0	None	305 ± 24	+
Cycloalkanes						
cyclopentane	None	83 ± 2	-	3 ± 2	196 ± 11	+
cyclohexane	None	101 ± 2	0	None	421 ± 17	+
Ethers						
dibutylether	None	59 ± 4	-	14 ± 13	347 ± 33	+
dipentylether	None	97 ± 2	0	None	211 ± 9	+
dihexylether	None	112 ± 4	0	None	113 ± 1	+
Esters						
ethyl heptanoate	None	78 ± 3	-	None	370 ± 34	+
ethyl octanoate	None	90 ± 1	-	None	285 ± 18	+
ethyl decanoate	None	98 ± 1	0	None	137 ± 2	+
Haloalkanes						
1-fluoropentane	None	76 ± 2	-	None	539 ± 35	+
1-fluorohexane	None	101 ± 1	0	11 ± 4	207 ± 13	+
1-fluoroctane	None	98 ± 1	0	None	182 ± 18	+
Ketones						
2-decaNone	None	81 ± 1	-	None	476 ± 52	+
2-undecaNone	None	98 ± 2	0	None	230 ± 16	+
2-dodecaNone	None	97 ± 3	0	None	325 ± 30	+
Sulfides						
1-(ethylthio)-hexane	None	87 ± 1	-	None	350 ± 57	+
1-(ethylthio)-octane	None	101 ± 1	0	None	120 ± 3	+
Thiols						
1-pentanethiol	None	85 ± 4	-	22 ± 8	466 ± 57	+
1-hexanethiol	None	102 ± 3	0	8 ± 2	290 ± 41	+

The % Direct Effect is the drug response without co-administration of the receptor agonist. The % Agonist Effect is the drug response with co-administration of agonist (glutamate and glycine for NMDA receptors; γ-aminobutyric acid for GABA_A_ receptors). The Drug Response denotes inhibition (**−**) for drug + agonist responses less than the control agonist peak, potentiation (+) for drug + agonist responses greater than the control agonist peak, and no response (0) for drug + agonist responses that differ by <10% from the control agonist peak.

**Figure 3:**
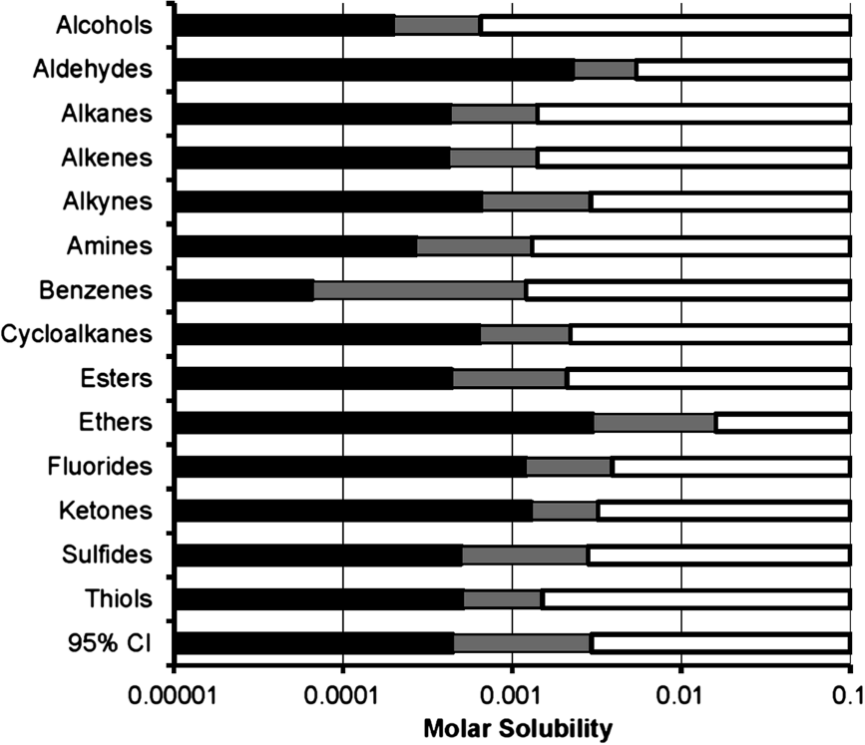
**Summary of receptor cut-off effects as a function of molar water solubility for compounds tested in Tables** 1 **and** 2 **.** For each hydrocarbon functional group, white bars represent compounds that modulate both GABA_A_ and NMDA receptors, and black bars represent compounds that modulate GABA_A_ receptors but have no effect on NMDA receptors at a saturating concentration. Intervening grey bars represent solubility values for which no data exist.

**Figure 4:**
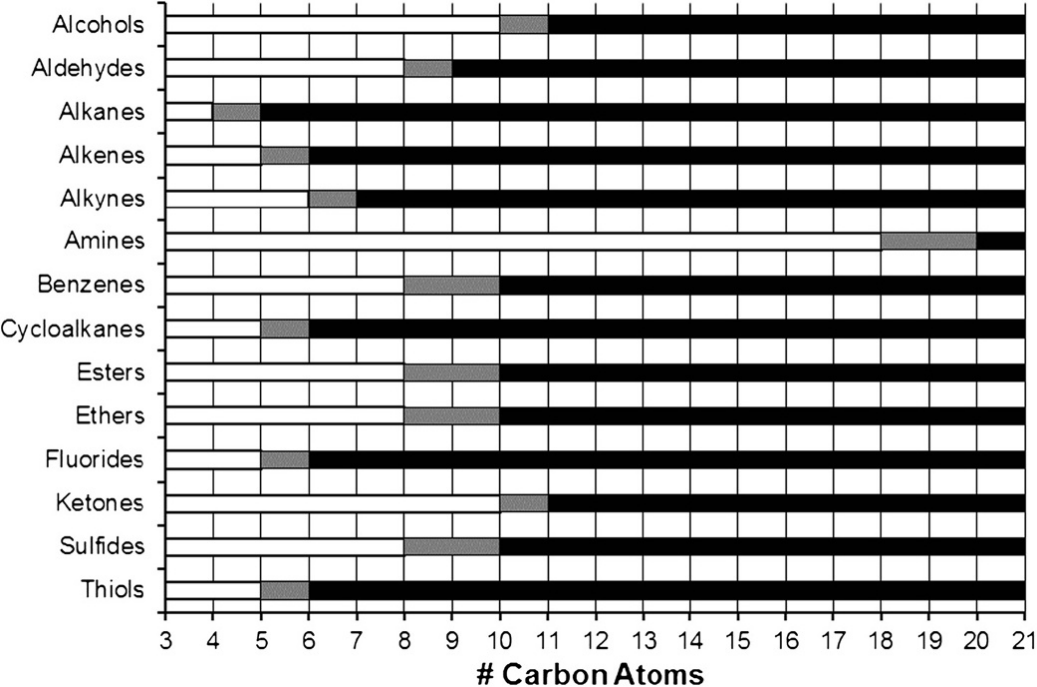
**Summary of receptor cut-off effects as a function of the number of drug carbon atoms for compounds tested in Tables** 1 **and** 2 **.** For each hydrocarbon functional group, white bars represent compounds that modulate both GABA_A_ and NMDA receptors, and black bars represent compounds that modulate GABA_A_ receptors but have no effect on NMDA receptors at a saturating concentration. Intervening grey bars represent solubility values for which no data exist. No receptor cut-off pattern is evident as a function of the number of drug carbon atoms.

**Figure 5:**
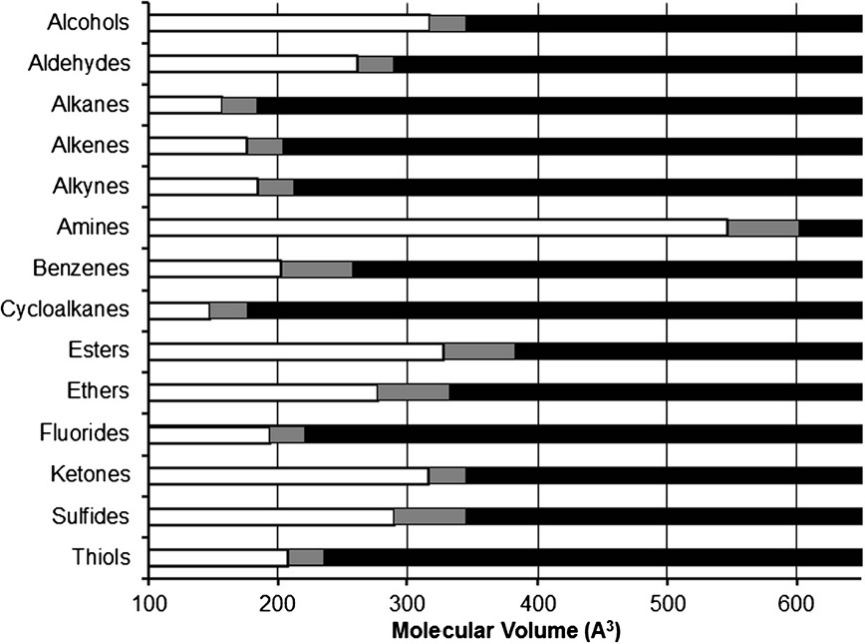
**Summary of receptor cut-off effects as a function of the calculated molecular volume of each drug for compounds tested in Tables** 1 **and** 2 **.** For each hydrocarbon functional group, white bars represent compounds that modulate both GABA_A_ and NMDA receptors, and black bars represent compounds that modulate GABA_A_ receptors but have no effect on NMDA receptors at a saturating concentration. Intervening grey bars represent solubility values for which no data exist. No receptor cut-off pattern is evident as a function of molecular volume.

In contrast, NMDA receptors currents were decreased by the shorter hydrocarbons within each functional group (Table 1), but lengthening the hydrocarbon chain eventually produced a null response—a cut-off effect. No direct hydrocarbon effects on NMDA receptor function were detected in the absence of glutamate and glycine agonist.The cut-off effect for NMDA receptor current modulation was associated with a hydrocarbon water solubility of 1.1 mM with a 95% confidence interval between 0.45 mM and 2.8 mM (Figure 3). More soluble hydrocarbons consistently inhibited NMDA receptor currents when applied at saturated aqueous concentrations, and hydrocarbons below this range had no appreciable effect on NMDA receptor function. Moveover, during the course of the study, water solubility was sufficiently predictive of an NMDA receptor cut-off so as to require identify and testing of only single pair of compounds bracketing this critical solubility value, as occurred with the alkenes, amines, cyclic hydrocarbons, and sulfur-containing compounds.Increasing hydrocarbon chain length decreases water solubility, but also increases molecular size. However, when graphed as a function of either carbon number (Figure 4) or molecular volume (Figure 5), the observed NMDA receptor cut-off effects show no consistent pattern. For example, the n-alkanes, 1-alkenes, and 1-alkynes show progressive lengthening of the hydrocarbon chain cut-off, presumably as a result of the increasing aqueous solubility conferred by the double and triple carbon bonds, respectively. There was also tremendous variation in molecular size of compounds exhibiting NMDA receptor cut-off. Alkanes exhibited NMDA receptor cut-off between butane and pentane, respectively 4 and 5 carbons in length, whereas the primary amines exhibited cut-off between 1-octadecanamine and 1-eicosanamine, respectively 18 and 20 carbons in length. As expected, the molecular volume of these compounds associated with NMDA receptor cut-off is also quite different, with the primary amine being over 3 times larger than the alkane.

## Discussion

NMDA receptor modulation is associated with an approximate 1.1 mM water solubility cut-off (Figure 3). In contrast, GABA_A_ receptors potentiated all study compounds; this may be because a GABA_A_ cut-off occurs at a lower water solubility value. Sequentially increasing hydrocarbon length to find a receptor cut-off effect potentially introduces confounding factors of carbon number and molecular volume that could in turn be responsible for the cut-off effect [35–38]. However, an aggregate comparison of cut-off values for all hydrocarbon groups as a function of carbon number (Figure 4) or molecular volume (Figure 5) shows no discernible pattern, suggesting that these physical properties are unlikely the primary limiting factors for drug-receptor modulation.

The correlation between molar water solubility and the NMDA receptor cut-off suggests hydrocarbons compete with water for amphipathic binding pockets within anesthetic-sensitive ion channels. Most inhaled anesthetics exhibit low-affinity binding on receptors as evidenced by generally large median effective concentrations for anesthesia—in the 230–290 μM range for isoflurane and halothane [39]—as compared to agents that exert narcotic effects via a singular or primary molecular targets. These specific interactions—exemplified by ketamine antagonism of NMDA receptors [12], etomidate agonism of GABA_A_ receptors [40], dexmedetomidine agonism of α_2_-adrenoreceptors [41], and morphine agonism of μ-opioid receptors [42]—typically require only a few μM or less of drug and are consistent with high affinity interactions to resulting in induced fit binding. Instead, volatile anesthetics likely bind to pre-existing pockets and surfaces on or within the protein [43]. Amphipathic pockets likely contain water molecules; when these are displaced by amphipathic drugs, fewer strong hydrophilic interactions and more hydrophobic interactions are possible with amino acid side chains in the cavity. We propose such nonspecific binding causes a change in pocket shape and, in consequence, the larger three-dimensional protein structure that affects channel gating or conductance.

Hydrocarbons act as hydrogen bond donors—or in the case of electrophiles, as hydrogen bond acceptors—with amino acid residues on anesthetic-sensitive receptors, resulting in displacement of water molecules from these binding pockets and alteration of protein function [44–46]. These low energy anesthetic-protein interactions are postulated to be enthalpically favorable since the displaced water molecules should be better able to hydrogen bond with like molecules in the bulk solvent rather than with amino acids [44, 46]. Halothane and isoflurane both have been shown to bind in water accessible pockets formed between α-helices in δ-subunits of the nicotinic acetylcholine receptor [47], a member of the 4-transmembrane receptor superfamily that includes the GABA_A_ receptor. Models of nicotinic acetylcholine receptors and GABA_A_ receptors further suggest that endogenous agonist or anesthetic binding might increase water accumulation in hydrophilic pockets and increase the number and accessibility of hydrophilic sites that are important for channel gating [48, 49]. However, molecules that are insufficiently water soluble may not be able to displace enough water molecules at enough critical sites in order to modulate channel function.

NMDA receptor modulation by inhaled anesthetics such as isoflurane, xenon, and carbon dioxide occurs—at least in part—at hydrophilic agonist binding sites [33, 50]. However, data from the present study show that less hydrophilic drugs—those with lower molar water solubilities—are still able to modulate GABA_A_ receptor currents even when NMDA receptor efficacy is lost. Since these receptors belong to different and phylogenetically distinct superfamilies, it seems likely that either the number of displaced water molecules required to effect modulation, the relative affinities of the hydrocarbon versus water molecule for a critical hydrophilic protein pocket, and/or the number of hydrophilic sites necessary for allosteric modulation should also be different between proteins. Simply stated, there is no reason to suppose that unrelated channels are likely to have conserved affinity constants for water within protein cavities capable of inducing an effect on ion conductance. As evidence, GABA receptors currents, but not those of NMDA receptors, can be modulated by compounds with molar aqueous solubilities much less than 1.1 mM. Presumably, the water dissociation constant in the NMDA receptor binding site is lower than that in the analogous GABA_A_ receptor binding site.

The locations within NMDA receptor subunits responsible for anesthetic-mediated modulation of protein function remain unresolved. However, it is likely that volatile anesthetics bind at multiple extracellular and transmembrane interfacial sites and cavities, as has been observed with nicotinic acetylcholine receptors [47, 51], *Gloeobacter* ligand-gated ion channels [52], and voltage-gated sodium channels [53]. Whether an anesthetic positively or negatively modulates an ion channel may be a function of competing drug interactions at different sites within the protein [54]. The protein cavities must to some extent be hydrophilic so that they are occupied by water in the native state. However, if these sites are too hydrophilic, then the energy necessary for a low-affinity drug to displace the water molecules becomes too great, requiring either very high drug concentrations that alter cell effects due to changes in serum osmolality or highly polar or charged drugs which, by virtue of these properties, are impermeable to the cell membrane and therefore cannot access critical transmembrane modulatory sites.

Although the present study assessed the association between water solubility and drug efficacy on anesthetic-sensitive ion channels, a relationship between solubility and potency may exist as well. For example, site-directed mutagenesis of the Ala825 residue on the M4 domain of the NR2A subunit, a region at which alcohols bind and can negatively modulate NMDA receptor currents [55, 56], affects potency of the slightly water-soluble hypnotic agent tribromoethanol. In fact, the hydrophobicity of the substituted amino acid negatively correlates with tribromoethanol potency as an NMDA receptor inhibitor [57]. Interestingly, except when replaced by the extremely hydrophobic tryptophan residue, which may change binding or access of water itself to the cavity, mutagenesis at this same site had no effect on the infinitely water-soluble anesthetic ethanol. Perhaps the small and highly polar ethanol molecule mimics many of the intramolecular forces, such as hydrogen bonding, of the water molecules it displaces within the binding site, thus extreme changes within the pocket are required to affect potency.

Water and lipid solubility also affect drug potency for wild-type receptors. Increasing chain length of straight-chain alcohols or diols increases hydrophobicity and is initially associated with increased inhibitory potency of NMDA receptors [58, 59]. Similarly, the magnitude of GABA_A_ receptor positive modulation in the present study tended to increase as hydrocarbon chains lengthened within any functional group. This is consistent with the Meyer-Overton prediction of increased anesthetic potency as a function of increasing hydrophobicity [60, 61]. The probability of a hydrocarbon passively entering a lipid cell membrane is parabolically related to hydrocarbon hydrophobicity; more hydrophobic molecules—up to a point—may be able to more easily access transmembrane modulatory sites [62]. However, drug solubility in the lipid membrane should not affect drug concentration in the amphipathic protein pocket, since the net energy involved in moving into and out of the lipid membrane are equal and opposite. The change in state—in this case, drug diffusion in a reversible path from perfusate to lipid membrane to protein pocket—is thermodynamically defined only by the initial state (free energy in perfusate) and final state (free energy of receptor binding), and the change in free energy is independent of the membrane path between these two states [63]. However, increasingly hydrophobic molecules should differ more in their intermolecular interactions with surrounding amino acid side chains compared to the water molecules they displaced. Therefore, if they can successfully access this amphipathic pocket, increasingly hydrophobic molecules may be capable of producing larger conformational changes in the protein and greater modulation of protein function. However, as molecules become even more hydrophobic and water solubility falls below the cut-off value, there are simply insufficient molecules in the aqueous phase to successfully compete with water at hydrophilic modulation or transduction sites on a receptor to alter its function. There is progressive loss of modulatory efficacy at sites with higher cut-offs that reduces the maximum drug-effect magnitude. Finally, when the drug water solubility becomes such that it is insufficient in concentration to out-compete the water in the lowest-cut-off site, the drug effect is reduced to zero.

Likewise, in the whole animal, this plausibly explains why transitional compounds and nonimmobilizers predicted by the Meyer-Overton correlation to produce anesthesia either have lower than expected potency or lack anesthetic efficacy altogether. As with the NMDA cut-off hydrocarbons presented in the present study, transitional compounds and nonimmobilizers all share a common property of low aqueous solubility [64]. Nonimmobilizers such as 1,2-dichlorohexafluorocyclobutane fail to depress GABA_A_-dependent pyramidal cells [65] or NMDA-dependent CA1 neurons [66] in the hippocampus, and likely lack these effects elsewhere in the central nervous system. With decreasing water solubility, there is differential loss of receptor effects—such as occurred with NMDA receptors versus GABA_A_ receptors in the present study. The anesthetic cut-off effect in whole animal models correlates with agent water solubility, and might be explained by the loss of one or more anesthetic-receptor contributions to central nervous system depression. Conversely, receptor molar water solubility cut-off values may be used to define those ion channels that are not essential for volatile anesthetic potency. Inhaled agents likely act via low affinity interactions with multiple cell receptors and ion channels to decrease neuronal excitability in the brain and spinal cord, but a loss or inadequate contribution from certain targets—perhaps GABA_A_ or glycine receptors—as water solubility decreases may render a drug a nonimmobilizer. Additionally, agents having a water solubility below the cut-off value for some anesthetic-sensitive receptors may also produce undesirable pharmacologic properties, such as seizures following the loss of GABA_A_ receptor modulation [67]. In contrast, NMDA receptors can contribute to immobilizing actions of conventional volatile anesthetics [3], but they are not as a general principle essential for inhaled anesthetic action since an agent like pentane does not modulate NMDA receptors—even at a saturated aqueous concentration (Table 2)—yet has a measurable minimum alveolar concentration [68, 69].

As shown in Figure 3, the different hydrocarbon series exhibit small variability about the 1.1 mM cut-off. Some variability is due simply to the lack of compounds of intermediate solubility within a functional group series. For example, pentanethiol inhibited NMDA receptors, whereas the 1-carbon longer hexanethiol did not (Table 2). This pre-cut-off thiol is nearly 3-times more soluble in water than its post-cut-off cognate; yet it is not possible to obtain a more narrowly defined cut-off delineation for 1-thiols. Even larger variability was observed with the dialkylbenzene series, to which 1 additional carbon was added to each 1- and 3-alkyl group. The solubility ratio between the NMDA antagonist 1,3-dimethylbenzene and its cut-off cognate 1,3-diethylbenzene is more than 18 (Table 2).

Variability about the molar water solubility NMDA receptor cut-off may also have arisen from the use of calculated, rather than measured, values for hydrocarbon molar water solubility. Aqueous solubility is difficult to measure accurately, particularly for poorly soluble substances. Calculated solubilities are more accurate for small uncharged compounds, but still can have an absolute error within 1 log unit [70]. However, even predicted values for nonpolar n-alkanes may show large deviations from experimental data as the hydrocarbon chain length increases [71].

Furthermore, the molar solubility values used in the present study were calculated for pure water at 25°C and at pH = 7.0. These were not the conditions under which drug-receptor effects were studied. Ringer’s oocyte perfusates contained buffers and physiologic concentrations of sodium, potassium, and chloride resulting in a 250 mOsm solution. The solubility of haloether and haloalkane anesthetic vapors vary inversely with osmolarity [72], as do the water-to-saline solubility ratio of benzenes, amines, and ketones [73]. The presence of salts could have caused overestimation of aqueous solubility for some compounds when using values calculated for pure water. Likewise, solubility is also temperature-dependent. Studies were conducted at 22°C; solubility of gases in water should be greater than values calculated at 25°C. In contrast, most solutes used in the present study have negative enthalpy for dissolution [74], so solubility should be decreased at the lower ambient temperature. The reverse should occur for exothermic solutions, as predicted by the Le Chatelier principle. As for hydronium ion concentration, the solubility of most study compounds is trivially affected at pH values between 7-to-8. However, hydrocarbons containing an amine group have pK_a_ values that are closer to physiologic pH, and the calculated aqueous solubility of 1-eicosanamine and 1-octadecanamine (Table 1) decreases by about 66% as pH increases from 7 to 8. Calculated molar water solubilities for the amines in this study were probably modestly overestimated at a physiologic pH equal to 7.4.

Only water solubility reliably predicted the NMDA receptor cut-off. Yet, molecular size and shape still likely influence this effect to some lesser degree. Most of the hydrocarbons examined in the present study had functional groups located on the 1- or 2-carbon position. However, the ethers were all 1,1′-oxybisalkanes; each member of the ether consisted of symmetrical 1-carbon additions to alkyl groups on either side of the oxygen atom (Table 1). Consequently, this electron-rich oxygen atom allowing hydrogen bonding with water molecules or amino acid residues with strong partial positive charges lies buried in the middle of the ether. For hydrocarbons with equivalent molar water solubilities, it may be more difficult for dialkyl ether to form hydrogen bonds in hydrophilic receptor pockets compared to a long primary amine (Table 1) that might more easily insert its nucleophilic terminus into the anesthetic-binding pocket while the long hydrophobic carbon chain remains in the lipid membrane. This may explain why ethers in this study appear to exhibit an NMDA cut-off that is slightly greater than hydrocarbons with other functional groups. Perhaps if a methyl-alkyl ether series were used instead of a dialkyl ether series, the apparent molar water solubility cut-off for this group would have been lower. Nonetheless, the cut-off variability is sufficiently small to allow *a priori* predictions of low-affinity hydrocarbon modulation of NMDA receptors. It remains to be shown whether other anesthetic-sensitive ion channels exhibit distinct cut-off effects that may also be predicted by a single physical property: molar water solubility.

## Conclusion

Cut-off responses for NMDA receptor inhibition by diverse hydrocarbons occurs when drug molar water solubility is less than approximately 1.1 mM. However, hydrocarbons having lower molar water solubilities are still able to potentiate GABA_A_ receptors. This finding supports a hypothesis that volatile compounds, such as inhaled anesthetics, access one or more amphipathic “pockets” within a protein, such as the NMDA receptor, to displace resident water molecules and induce a conformational change. Volatile anesthetics and other compounds with insufficient molar water solubility can never achieve sufficient concentration at the amphipathic pocket site to displace water and modulate protein function—even when such compounds are administered at a saturated aqueous phase concentration.
